# Physical Activity Before, During, and After the School Day and Its Impact on Positive Psychological Variables: A Systematic Review and Practical Guide

**DOI:** 10.3390/healthcare14162625

**Published:** 2026-08-19

**Authors:** Pablo Ramírez-Espejo, José Luis Solas-Martínez, Manuel J. De la Torre-Cruz, Alberto Ruiz-Ariza

**Affiliations:** 1 Department of Didactic of Musical, Plastic, and Corporal Expression, University of Jaén, 23071 Jaén, Spain; pramirez@ujaen.es (P.R.-E.); arariza@ujaen.es (A.R.-A.); 2 International Network for Research in Education Law (RIIDE), 23071 Jaén, Spain; majecruz@ujaen.es; 3 Ibero-American Network for Research and Teaching in Physical Activity Sciences (RIIDCAF), 23071 Jaén, Spain; 4 International Scientific Association for Research in Physical Activity, Health, and Learning (ACIAFSA), 23071 Jaén, Spain; 5 Department of Psychology, University of Jaén, 23071 Jaén, Spain

**Keywords:** physical education, school-based interventions, timing of physical activity, children and adolescents, positive psychology

## Abstract

**Background/Objectives**: Positive psychological constructs such as psychological well-being, resilience, subjective vitality, and mental toughness are fundamental to students’ emotional adjustment and overall development. Physical activity (PA) is a modifiable factor known to enhance these variables; however, the influence of its timing throughout the day remains underexplored. This systematic review aimed to examine the effects of PA on psychological well-being, resilience, subjective vitality, and mental toughness in children and adolescents, considering the timing of implementation—before, during, or after school hours—and the specific characteristics of the activities performed. **Methods**: Following the PRISMA guidelines, a comprehensive search was conducted in Scopus, PubMed, ERIC, and Web of Science, identifying 29 studies published between 2014 and 2026, comprising 12 cross-sectional and 17 longitudinal studies. **Results**: Overall, PA was positively associated with all four psychological variables across different times of the day. PA before school, such as active commuting and active starts, was primarily associated with higher in well-being and vitality. During-school interventions, particularly structured programs such as active breaks and cooperative games, were associated with better resilience, emotional self-regulation, and prosocial behavior. After-school PA, especially organized and voluntary activities, was associated with higher resilience and mental toughness. The effects varied according to the intensity, structure, social context, and student role, with moderate-to-vigorous intensities and cooperative formats producing the most consistent benefits. **Conclusions**: In conclusion, PA appears to exert a beneficial influence on positive psychological development, with variations depending on the time and mode of practice. Schools, particularly through Physical Education programs, constitute a key context for promoting students’ emotional, motivational, and behavioral health.

## 1. Introduction

In the current educational context, fostering positive psychological development among young people has become a priority for promoting their mental and personal well-being [1,2]. Positive psychology, understood as the scientific approach that examines the strengths, resources, and processes enabling individuals to thrive and develop optimally [3], offers a relevant framework for this purpose. Some of the key factors to consider in consolidating positive psychological functioning during adolescence include psychological well-being, resilience, subjective vitality, and mental toughness, as these are directly related to better emotional, motivational, and behavioral adjustment in educational settings [4]. Recent evidence indicates that the deterioration of these psychological variables negatively affects school adaptation, stress regulation, academic motivation, and students’ ability to effectively cope with everyday challenges within the educational environment [5,6]. Moreover, these dimensions not only reflect students’ general psychological state but also serve as strong predictors of their capacity to modify behaviors and assimilate new learning, even when such learning challenges their prior beliefs [7].

Psychological well-being is a positive state of physical and mental balance associated with perceived security, competence, and social connectedness [8]. During adolescence, it is linked to life satisfaction, academic motivation, and interpersonal relationships [9,10,11]. Resilience, defined as the ability to adapt positively to adversity [12], becomes particularly relevant during this developmental stage due to increasing social and emotional demands [13]. Higher resilience is associated with lower anxiety and academic burnout, as well as greater school engagement and goal persistence [13,14]. Subjective vitality refers to the internal sense of energy, enthusiasm, and motivation experienced in daily life [15]. As a key indicator of eudaimonic well-being, it is associated with happiness, self-actualization, perceived health, and intrinsic motivation [16,17]. In adolescents, vitality is positively related to life meaning, psychological development, coping resources, and peer relationships, supporting socio-emotional adaptation in school contexts [18,19,20,21]. Closely related, mental toughness is a meta-skill that enables effective management of personal resources under pressure or adversity [22]. Characterized by self-confidence, emotional stability, and sustained focus [23], higher mental toughness is associated with better adaptation to academic and personal challenges, fewer psychological symptoms, and greater overall well-being during adolescence [24,25,26].

These four constructs together form a comprehensive framework of positive psychology in adolescent populations, whose development is conditioned by various behavioral and psychosocial factors [3]. Among these, one factor that has gained increasing attention due to its direct relationship with these dimensions is the adoption of an active lifestyle, strongly supported by empirical evidence demonstrating multiple benefits for physical, mental, and social health [27]. Regular physical activity (PA) has been established as a powerful promoter of psychological well-being, fostering greater emotional stability and enhanced coping capacity when facing daily challenges [1]. Recent studies highlight that consistent participation in PA contributes to reducing symptoms of anxiety and depression while promoting a positive mood and greater life satisfaction [28,29,30,31].

Moreover, PA has been associated with social, academic, and emotional benefits, serving as an effective means of improving social integration, enhancing academic performance, and supporting balanced psychological development [32,33,34,35]. These effects can be explained through physiological mechanisms, such as increased cerebral blood flow and the release of well-being-related neurotransmitters like serotonin, dopamine, and endorphins, and psychological mechanisms, including improved self-efficacy, body perception, emotional regulation, and reduced perceived stress [10]. More specifically, PA has been shown to enhance resilience by providing experiences of personal growth and adaptive coping strategies [36,37], to foster subjective vitality by increasing perceived energy, enthusiasm, and engagement in everyday activities [18,38] and to contribute to the development of mental toughness through improvements in self-confidence, emotional control, and psychological endurance [25,26,39].

Within this framework, several studies have begun exploring how the time of day when PA is practiced may modulate its effect on different dimensions of psychological development [40,41]. Beyond the amount and type of PA, increasing attention has been given to the timing of physical activity, as the physiological, cognitive, emotional, and social context in which PA is performed may influence its potential psychological effects. From this perspective, PA performed before, during, and after school may provide different opportunities for emotional activation, social interaction, self-regulation, and autonomous engagement. First, engaging in PA before the school day may facilitate physical–mental activation, emotional well-being, and social interaction among students [42,43]. Activities such as active commuting to school or “active starts” (brief periods of PA integrated at the beginning of the school day or lessons) have been shown to promote a mood state conducive to learning and socialization during classes [44,45,46].

Second, during the school day, when adolescents spend long hours seated [47], incorporating active breaks, physically active lessons, or structured recess not only contributes to the movement necessary for health but also improves classroom emotional climate and encourages more cooperative peer relationships [48,49,50]. Furthermore, physical education (PE) lessons represent a privileged context for intentionally addressing psychological well-being [38,51,52]. Activities such as cooperative games, problem-solving exercises, or high-intensity circuits can promote self-efficacy, emotional self-regulation, and intrinsic motivation [38,53,54].

Finally, after-school PA generally occurs in more voluntary and recreational contexts, involving more autonomous and enjoyable motivational engagement [55,56]. This freer, playful nature encourages adolescents to participate out of personal interest, reinforcing enjoyment, self-determination, and long-term adherence [32]. Unlike in-school PA, which follows curricular criteria, after-school activities have been linked to distinct benefits, such as higher resilience development through less structured challenges requiring self-regulation and personal adaptation [57]. Moreover, team sports practiced in clubs, community centers, or gyms foster interpersonal and emotional skills essential for psychological well-being and strengthen mental toughness by exposing participants to situations involving pressure, sustained effort, and collaboration [58,59].

Considering other relevant factors, several covariates may influence the relationship between PA and the development of well-being, resilience, subjective vitality, and mental toughness. During primary and secondary education, corresponding with the onset of adolescence, a progressive decline in PA levels is observed, attributed, among other things, to the increased use of technology and social media both associated with sedentary lifestyles and a higher risk of depressive symptoms and low mood [60,61]. Similarly, variables such as age, sex, or socioeconomic status may significantly affect the perception, accessibility, and benefits derived from PA [62]. For instance, inequalities in access to sports facilities or organized activities according to sociocultural context can directly impact students’ psychological well-being [63].

In this regard, the complexity of contextual and personal factors surrounding PA underscores the need to analyze not only whether PA is practiced, but when and how it occurs throughout the day. Although previous systematic reviews have consistently demonstrated that physical activity benefits children’s and adolescents’ mental health and psychological well-being, these reviews have generally focused on overall physical activity levels, specific intervention modalities, or isolated psychological outcomes. To our knowledge, no previous systematic review has comprehensively examined whether the timing of physical activity implementation (before, during, or after school hours) differentially influences positive psychological constructs such as psychological well-being, resilience, subjective vitality, and mental toughness. Therefore, the present review addresses this specific gap by integrating evidence according to both the timing and characteristics of school-related physical activity interventions [64,65]. To our knowledge, no previous systematic review has integrated these four outcomes according to the timing and characteristics of PA in school-aged populations. Therefore, this review addresses a specific gap in the literature by considering both when PA is performed and how its characteristics may influence positive psychological development. Based on this gap, the objective of the present systematic review was to analyze the influence of PA on these four positive psychology constructs in children and adolescents, considering both the time of implementation (before, during, or after school hours) and the specific characteristics of the PA interventions.

## 2. Materials and Methods

This systematic review was conducted and reported in accordance with the Preferred Reporting Items for Systematic Reviews and Meta-Analyses (PRISMA 2020) Statement [66], in accordance with the methodological structure adopted in previous reviews on PA and school health [40,67,68,69]. The completed PRISMA 2020 checklist is provided as Supplementary Material (Table S1). The review protocol was prospectively registered in the International Prospective Register of Systematic Reviews (PROSPERO) under registration number CRD420261472150. The review methodology was established a priori before the literature search was conducted, and no amendments were made to the protocol during the review process. A comprehensive search was performed across four major scientific databases: SCOPUS, ERIC, Web of Science, and PubMed. These databases were selected to provide complementary coverage of biomedical and health sciences (PubMed), multidisciplinary scientific literature (Scopus and Web of Science), and educational research (ERIC). Specialized databases such as PsycINFO and SPORTDiscus were not included, which may have resulted in the omission of some relevant studies and is acknowledged as a limitation of the search strategy.

The publication period for the studies included ranged from January 2014 to May 2026. Table 1 presents the main search terms and Boolean combinations used: (1) Physical activity (physical activity academic lessons, physical active program, physically active classes, physical education, active lesson break, active break, active pauses, recess, recreation, cooperative games, active movement, active displacement, active pre-school programs, extracurricular activities, after school activities, extracurricular sport); (2) Positive psychological variables (well-being, resilience, subjective vitality, mental toughness); (3) Children and adolescents (children, child, childhood, students, adolescent, adolescents, school, secondary school).

### 2.1. Selection Criteria

Articles retrieved during the search were selected according to the following criteria: (1) the study reported the time of day when PA was performed (before, during, or after school hours); (2) the sample consisted of children or adolescents aged 6–18 years; (3) The study employed a cross-sectional or longitudinal design; (4) the article was a full-text publication in a peer-reviewed journal; (5) the article was written in English or Spanish. The language restriction was applied because English and Spanish are the languages in which the review team has full scientific proficiency, ensuring accurate study selection, methodological quality assessment, and data extraction while minimizing potential translation bias. English was selected because it is the predominant language of scientific publication, whereas Spanish was included due to the substantial contribution of Spanish-speaking countries to school-based physical activity research. (6) Ethnic origin was not considered an exclusion criterion.

### 2.2. Data Extraction and Reliability

The search process was conducted by three independent reviewers (R1, R2 and R3), who screened the titles and abstracts of all retrieved records for eligibility articles. Potentially relevant studies were subsequently assessed in full text according to the predefined eligibility criteria. Disagreements regarding study eligibility were resolved through discussion and consensus. Although inter-reviewer agreement was not quantified using a formal statistical coefficient, the independent screening process and consensus procedure were applied to enhance the reliability and transparency of study selection. Data were then extracted from each included study on the author, title, objective, sample size, participants’ age, country, methodological design, measurement instruments, confounding factors considered, main results, and conclusions. The included studies were also examined in depth according to the thematic areas addressed in the review.

### 2.3. Methodological Reporting Assessment

According to a recent review, only about one-third of systematic reviews in the educational field include tools for assessing the quality of the analyzed studies [70]. Based on this evidence, the present review implemented a methodological reporting assessment using a six-item checklist adapted from previous systematic reviews [40,71], alongside the specific selection criteria established for this investigation (see Table 2). Each item was rated as follows: 2 = fully reported, 1 = partially reported, and 0 = not reported or unclear. The checklist consisted of six items referring to study population and participant characteristics, context and physical activity timing, measurement instruments, study design, control of potential confounding factors and reporting of results. The total checklist score ranged from 0 to 12 and was used only to describe checklist performance within the present review.

Scores of 9–12, 5–8, and 0–4 were retained as operational checklist score ranges for descriptive purposes within this review. These categories were not derived from or intended to represent externally validated thresholds of internal validity, methodological rigor, certainty of evidence, or risk of bias. Similarly, eligibility characteristics, including participant age, publication in a peer-reviewed journal, and reporting of PA timing, were considered inclusion criteria and were not interpreted independently as indicators of methodological quality or rigor.

### 2.4. Methodological Reporting Assessment and Checklist Performance

The methodological reporting characteristics of the included studies were independently assessed by three reviewers using a standardized six-item checklist adapted from previous systematic reviews in the fields of physical activity and school health. The checklist evaluated six methodological domains: study population and participant characteristics, intervention context and physical activity timing, measurement instruments and outcome assessment, study design, control of potential confounding factors, and reporting of results. Disagreements were resolved through discussion until consensus was reached. Although established design-specific risk-of-bias tools (e.g., RoB 2 or ROBINS-I) are available, they were not applied because the included evidence comprised heterogeneous cross-sectional and longitudinal designs, interventions, and outcome assessments. A common methodological quality checklist was therefore considered appropriate for characterizing the overall evidence base and identifying potential methodological limitations across studies. Importantly, the checklist was not considered a formal risk-of-bias instrument and should therefore not be interpreted as providing a validated assessment of internal validity. These limitations were considered when interpreting the findings.

### 2.5. Reporting Bias and Certainty of Evidence

Because of the heterogeneity of study designs, interventions, outcome measures, and reported results, a formal assessment of reporting bias and certainty of evidence (e.g., GRADE) was not performed. Therefore, findings were synthesized narratively following the PRISMA 2020 recommendations.

## 3. Results

**Figure 1:**
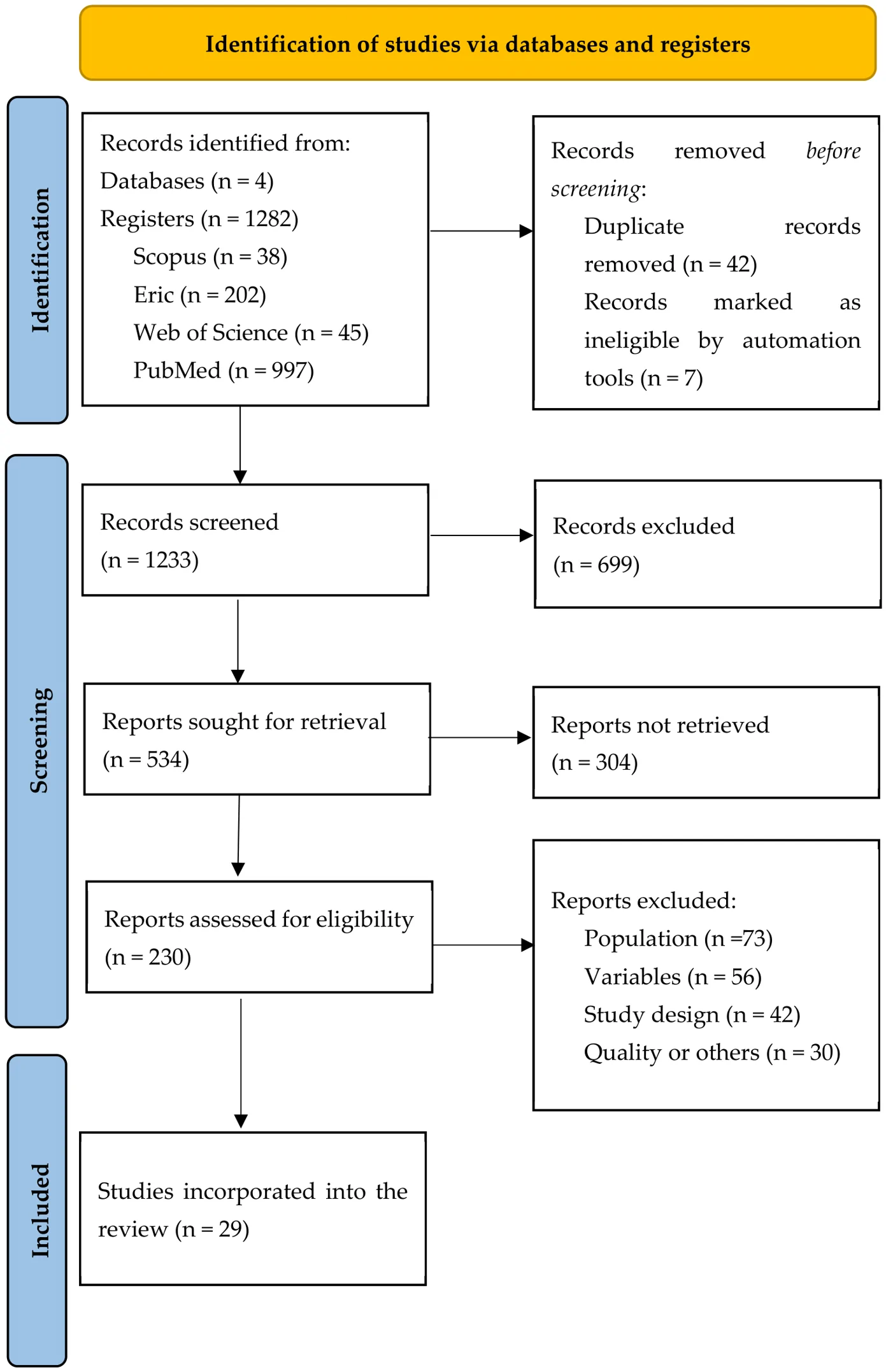
PRISMA 2020 flow diagram.

Figure 1 presents the PRISMA flow diagram illustrating the study selection process. The included studies showed substantial heterogeneity in methodological design, PA characteristics and timing, intervention duration and intensity, psychological outcome measures, and follow-up periods. Therefore, the findings were synthesized narratively, and direct comparisons of intervention effectiveness should be interpreted with caution. The initial search yielded a total of 1282 records, of which 1233 were retained after removing duplicates and unreadable entries. Subsequently, 699 studies were excluded after title screening, and an additional 304 were eliminated following a preliminary abstract review because could not be obtained despite the retrieval procedures applied. These procedures included database full-text links, institutional library access, publisher platforms, and/or searches for legally accessible full-text versions. The 304 non-retrieved reports corresponded to publications for which the full-text report could not be obtained during the retrieval stage, including those for which no accessible full-text version could be located. Thus, 56.9% of the reports sought for retrieval could not be obtained, representing a potential limitation to the completeness of the evidence base that should be considered when interpreting the findings. Finally, after applying inclusion criteria related to population characteristics, measured variables, methodological design, and study quality, 29 studies were included in the systematic review. Among these, 12 studies (41.38%) employed a cross-sectional design, and 17 studies (58.62%) employed a longitudinal design. In terms of checklist performance, 28 of the 29 studies obtained scores within the 9–12 operational range, whereas one study obtained a score within the 5–8 range. These score ranges describe the performance of the studies on the checklist items and should not be interpreted as evidence of high or low internal validity or risk of bias. The predominance of scores within the upper operational range should nevertheless be interpreted cautiously, because the checklist was primarily intended to characterize methodological reporting and selected study characteristics rather than to provide a discriminative or validated assessment of internal validity or risk of bias.

The review encompassed data from a total of 99,947 students aged between 6 and 18 years. Of these, 8661 were from primary education (6–12 years), 90,732 from secondary education (13–18 years), and 554 participants had unspecified or mixed age ranges. Regarding sample size, four studies included fewer than 100 participants [72,75,90,92]; thirteen studies involved between 101 and 1000 participants [37,38,46,50,55,57,74,80,81,82,87,88,93]; and twelve studies included samples larger than 1001 participants [44,73,76,77,78,79,83,84,85,86,89,91]. The studies covered 13 different countries of origin: five studies were conducted in Spain, four in China, four in Australia, three in England, two in Turkey, two in Norway, two in Denmark, and one each in Panama, Austria, Lithuania, Ireland, Switzerland, and Poland. The main characteristics of the analyzed studies are summarized in Table 3.

Notably, the findings were not uniformly positive across all studies. Some investigations reported null or weak associations between PA and psychological outcomes, including studies in which PA or moderate-to-vigorous PA was not significantly associated with mental well-being or global mental health symptoms. Such discrepancies may reflect differences in study design, participant characteristics, baseline psychological status, intervention duration and intensity, outcome measurement instruments, and the extent to which potential confounders were considered. These findings indicate that the overall favorable pattern should not be interpreted as evidence of uniform effects across populations or psychological outcomes.

### 3.1. Influence and Effects of Physical Activity Before School Day on Positive Psychology Variables

With respect to PA performed before school hours, the scientific literature remains limited, with only three studies identified. Two cross-sectional studies reported associations between active commuting to school (walking or cycling) and higher levels of psychological well-being and subjective happiness, as well as lower emotional distress [44,86]. A longitudinal study implementing a four-week intervention reported that participants in the experimental group exhibited a significant increase in well-being compared to controls [46].

### 3.2. Influence and Effects of Physical Activity During School Day on Positive Psychology Variables

A total of 12 studies examined the impact of PA performed during school hours on well-being, resilience, subjective vitality, and mental toughness. Of these, two employed cross-sectional designs and ten used longitudinal designs. Findings indicate that engaging in structured PA formats, such as cooperative games, active recess, or integrated movement sessions within lessons, were consistently associated with improvements in resilience, self-efficacy, subjective vitality, and adaptive behavior. Guillamón et al. [80] found that higher physical fitness levels were related to lower hyperactivity and greater prosocial behavior, whereas Li et al. [83] demonstrated that PA predicted resilience via self-efficacy and the satisfaction of basic psychological needs. School-based interventions such as multicomponent programs, park-based activities, or initiatives like Burn 2 Learn showed positive effects on vitality, general well-being, and task performance [38,88,92]. Additionally, martial arts and sport-based interventions were found to be more effective than traditional therapies in enhancing mental toughness, self-confidence, and physical self-efficacy [50,72].

The intensity of exercise emerged as a key factor influencing the magnitude of these effects. High-intensity programs consistently improved self-esteem, emotional well-being, social support, and sociability, with particularly strong effects among female students [84,87,91]. However, not all findings were conclusive: some studies found no clear relationship between moderate-to-vigorous PA and overall mental well-being, although specific benefits such as reduced anxiety and depressive symptoms were reported [76,90].

### 3.3. Influence and Effects of Physical Activity After School Day on Positive Psychology Variables

Fourteen studies analyzed the impact of after-school PA on well-being, resilience, subjective vitality, and mental toughness, of which eight were cross-sectional and six longitudinal. Cross-sectional studies generally reported positive associations between extracurricular PA and psychological outcomes among children and adolescents. Participation in organized sports was positively associated with higher well-being, resilience, and mental toughness [55,57,77,82]. Greater PA intensity was correlated with higher sleep quality, lower depressive symptoms—particularly among girls—and higher levels of personal competence and family cohesion [85]. Moreover, coping strategies were found to mediate the relationship between PA and resilience, emphasizing the value of unstructured active play [81]. Team sports participation was also linked to greater resilience compared with sedentary peers [79,89].

Longitudinal evidence further demonstrated that regular PA predicts sustained improvements in resilience and well-being [37,73,93]. Interventions involving organized sports combined with social activities yielded positive effects on mental well-being after several weeks [75]. Additionally, vigorous-intensity PA increased positive affect up to a threshold of approximately 36 min per day, after which the benefit plateaued or slightly declined [78]. Finally, programs incorporating psychological coaching were found effective in enhancing well-being and healthy behavior patterns [74].

## 4. Discussion

The present systematic review aimed to examine the influence of different types of PA conducted before, during, and after school hours on psychological well-being, resilience, subjective vitality, and mental toughness among students aged 6 to 18 years. A total of 29 studies, employing cross-sectional and longitudinal designs and published between January 2014 and May 2026, were included. The findings indicate that PA, particularly when implemented in structured formats (e.g., cooperative games, team sports, or martial arts), at moderate-to-vigorous intensity levels, and at different times of the day, is generally associated with improvements in at least one of the psychological variables analyzed. Specifically, after-school and during-school interventions showed relatively consistent favorable associations with resilience, subjective vitality and mental toughness, whereas some before-school-based interventions also reported improvements in mental toughness and overall well-being. However, the results were not uniform across studies, as some reported null effects, particularly regarding general psychological well-being, when the intensity or duration of PA was insufficient or inadequately prescribed. This overall positive pattern should nevertheless be interpreted cautiously because the included studies differed substantially in design, intervention characteristics, duration, intensity, outcome measures, and contextual conditions. Consequently, the consistency observed in the direction of the findings does not necessarily imply comparable effect magnitudes across studies.

Studies analyzing PA before school hours generally reported favorable associations with well-being, subjective vitality, and emotional self-regulation, although evidence regarding resilience and mental toughness was more limited. Specifically, three studies evaluated structured morning interventions, such as active commuting, active start programs, or combined routines, and reported improvements in mood, attention, enthusiasm, and readiness to learn [44,46,86]. Additional favorable associations were reported in concentration and on-task behavior, especially when the activities included cooperative dynamics or high-intensity bouts performed prior to class time, consistent with the findings of White et al. [94]. These positive effects appear to be explained by neurophysiological and motivational mechanisms that optimize physical–mental activation at the beginning of the day, facilitating better emotional and cognitive adaptation [95]. Nonetheless, no study identified significant improvements in resilience or mental toughness exclusively linked to morning PA. This absence suggests that, while pre-class PA may facilitate a positive emotional climate and general well-being, its influence on more complex personal resources, such as resilience or mental toughness may require longer-term or differently focused interventions [86,96].

In contrast, PA during the school day was generally associated with favorable patterns across several positive psychology variables. Interventions implemented through active lessons, structured recess, active breaks, or specific exercise programs demonstrated improvements in self-efficacy, on-task behavior, emotional self-regulation, and general mood [50,72,80,83,84,87,91,92]. These interventions were also associated with reductions in anxiety and depressive symptoms, hyperactivity, and classroom conflicts, as well as increases in prosocial behavior [97,98,99,100].

The beneficial effect of PA on resilience and mental toughness appears to be partially mediated by motivational variables, such as self-efficacy and the satisfaction of basic psychological needs [83]. Moreover, cooperative approaches and group-based activities (e.g., games or problem-solving tasks) seem to have a stronger impact on subjective well-being and self-confidence than individual or low-intensity activities [50,72,88]. However, some studies [76,101] did not find a clear relationship between moderate-to-vigorous PA and mental well-being, although they reported indirect benefits such as better sleep, reduced anxiety, and lower emotional distress. In addition, the absence of PA was consistently linked to a higher risk of emotional problems [97,98,99,100].

Several contextual factors including teaching style, motivational climate, socioeconomic status, school type, and cultural expectations may also modulate the psychological impact of school-based PA [4,9,11,102]. Evidence indicates that autonomy-supportive teaching that fosters intrinsic motivation and active student participation generates greater emotional benefits and reduces the risk of frustration or demotivation [88,103]. Recent research further emphasizes the importance of cooperative, rather than overly competitive, instructional approaches to promote social cohesion, emotional stability, and school adjustment [14,31,104,105].

Additionally, physical education teachers recognize the potential of PA as a pedagogical tool to foster psychological well-being, resilience, and subjective vitality among students, thereby supporting greater learning readiness and a more positive attitude toward academic and personal challenges [6,106].

Finally, PA after school hours produced beneficial effects on well-being, resilience, subjective vitality, and mental toughness. Specifically, extracurricular activities, especially those that were structured, voluntary, and recreational, were associated with greater self-efficacy, emotional strength, and psychosocial adjustment [11,37,57,73,79,89,93]. These positive effects appeared to depend on the type of activity, its group or individual nature, frequency, and the role played by the student (e.g., leader, substitute, or active team member) [107,108]. Participation in team sports or collective activities enhanced socio-emotional skills such as cooperation, social support, sense of belonging, and emotional coping, while also improving mood and reducing depressive symptoms [81,109,110]. Conversely, individual PA, such as martial arts or combat sports (e.g., judo or wrestling), also strengthened mental toughness, emotional self-regulation, and self-confidence, though it may elicit higher stress levels if competitive pressure is not properly managed [111,112,113,114]. Furthermore, the psychological benefits of extracurricular PA were amplified when the activities were well structured and supervised, contributing to the development of resilience, family cohesion, and personal competence [74,82,85]. Improvements were also observed in variables related to overall well-being, such as school enjoyment, positive learning attitudes, and sleep quality [77,115]. In line with this evidence, engaging in PA at the end of the school day, typically lasting at least one hour and performed at moderate-to-vigorous intensity, was found to facilitate circadian rhythm regulation and reduce nocturnal cognitive hyperactivation, thereby contributing to more efficient, deeper, and restorative sleep [116,117].

### 4.1. Mechanisms Underlying Effects of Physical Activity on Positive Psychological Variables

From a neurophysiological perspective, PA enhances cerebral blood flow and improves oxygenation in key cortical regions involved in emotional regulation and executive functioning [1]. These mechanisms may also help explain why cooperative and socially structured activities showed relatively consistent benefits across several outcomes in the present review. In particular, opportunities for shared goals, peer interaction, feedback, and perceived competence may simultaneously support self-efficacy, social belonging, emotional regulation, and resilience. However, these mechanisms should be regarded as plausible explanatory pathways rather than causal mechanisms directly demonstrated by the included studies. Muscle contraction during exercise stimulates the release of myokines and other signaling molecules that promote the secretion of neurotransmitters such as dopamine, serotonin, and norepinephrine, all of which are associated with emotional balance, motivation, and stress reduction [5,10,118,119,120,121].

Moreover, physical exercise induces the release of endocannabinoids and endogenous opioids (e.g., endorphins, enkephalins, and dynorphins) that act on the central nervous system, promoting a general state of well-being. This process also modulates kynurenine metabolism, reducing its neurotoxic effects and favoring a more neuroprotective biochemical profile, which contributes to decreased depressive and anxiety symptoms [118,122,123]. Structurally, PA has been shown to foster neuroplasticity and increase gray matter volume in regions such as the hippocampus and prefrontal cortex, which are implicated in decision-making, emotional self-regulation, and planning. From a psychosocial standpoint, participation in group-based PA enhances social belonging, cohesion, and self-efficacy, key factors in the development of psychological well-being and resilience within the school context [124,125,126]. Such positive environments promote interpersonal relationships, self-confidence, and perceived social support, all of which contribute to greater life satisfaction and a more adaptive emotional attitude toward academic and personal challenges [127,128].

Taken together, these neurobiological and psychosocial mechanisms operate in a synergistic manner, promoting enhanced emotional regulation, coping capacity in the face of stressful situations, and the development of a healthier, more balanced school environment.

### 4.2. Practical Recommendations

The present systematic review suggests that PA performed before, during, and after the school day exerts a positive, sustained, and differentiated impact on psychological well-being, resilience, subjective vitality, and mental toughness among children and adolescents [12,29,30]. However, the available evidence is heterogeneous and the effectiveness of these interventions depends on multiple factors related to planning, intensity, the group or individual nature of the activity, the school context, and the developmental characteristics of the students involved [7,28,32].

Across the included studies, several activity characteristics were repeatedly represented among interventions or activity formats reporting favorable psychological outcomes, including structured programs, regular participation, moderate-to-vigorous intensity, active participation, cooperation, and opportunities for emotional self-regulation [29,32]. However, these characteristics should be considered provisional, evidence-informed considerations rather than established components of an effective intervention. Some included studies suggest that interventions incorporating playful components, cooperative tasks, and student autonomy may be associated with more favorable psychological outcomes; however, the absence of a meta-analysis and formal certainty-of-evidence assessment prevents firm conclusions regarding comparative effectiveness [7,30]. At the same time, these interventions should be adapted to the students’ developmental level, taking into account their cognitive and affective changes across educational stages [12,28]. Therefore, although the following recommendations are general and applicable to both children and adolescents, it is essential to adjust the difficulty and intensity of activities according to the students’ individual characteristics in order to optimize their effectiveness.

The activities presented in Table 4 were derived from the intervention characteristics and activity formats reported in the included studies and subsequently organized by the authors to provide practical examples according to timing, intensity, duration, and reported psychological outcomes. They should be interpreted as illustrative, provisional, evidence-informed examples rather than standardized intervention protocols or prescriptions of demonstrated effectiveness. Within this framework, Table 4 summarizes the main practical recommendations, organized according to the time of day when PA is implemented (before, during, or after school hours). The table provides specific guidance regarding the type of activity, intensity, duration, and expected outcomes, with the aim of promoting healthier, more equitable, and emotionally enriching school environments [30].

### 4.3. Limitations and Strengths

This review presents several limitations that should be considered when interpreting its findings. First, a smaller number of studies were identified that specifically examined the psychological effects of PA performed before the start of the school day, compared to those focused on PA conducted during or after school hours. This imbalance limits the ability to draw firm conclusions regarding the relative effectiveness of morning interventions compared to activities carried out at other times of the day. In addition, some of the included studies did not account for key covariates that may influence the positive psychology variables analyzed in this review, such as socioeconomic status, family support, or academic workload, which could play a significant mediating role in the relationship between PA and its psychological effects. It is also important to note that the heterogeneity of interventions, methodological designs, and measurement instruments used across studies may hinder direct comparison of results and limit the generalizability of conclusions. The evidence base included heterogeneous study designs, with a substantial proportion of cross-sectional studies, limiting the ability to establish temporal directionality or causal relationships, also, the methodological quality checklist used in this review should not be interpreted as a formal validated risk-of-bias assessment, and no formal certainty-of-evidence grading was performed. A further limitation is the relatively high proportion of reports that could not be retrieved at the full-text stage (304 of 534 reports sought for retrieval). Although the available retrieval procedures were applied, the absence of these reports may have resulted in the omission of potentially relevant evidence and should therefore be considered when interpreting the completeness of the review.

A further methodological limitation is that inter-reviewer agreement was not quantified using a formal statistical coefficient such as Cohen’s κ. Although study screening was conducted independently by three reviewers and disagreements were resolved through consensus, the original screening records did not allow for reliable retrospective reconstruction of a pre-consensus agreement coefficient. Furthermore, the absence of a meta-analysis limits the estimation of pooled effect sizes and the quantitative assessment of between-study heterogeneity. Consequently, the conclusions of this review should be interpreted as reflecting the overall direction and consistency of the available evidence rather than precise estimates of intervention effects. Despite these limitations, the present review offers an original contribution by classifying scientific evidence according to the time of day at which PA is performed (before, during, and after school hours). This approach allows for the identification of differential patterns in the effects of PA on psychological well-being, resilience, subjective vitality, and mental toughness among school-aged populations. Among its strengths, this review combines cross-sectional and longitudinal studies, thereby providing an integrated understanding of both immediate and sustained effects. Furthermore, it presents evidence-based practical recommendations, organized by time of day, offering valuable guidance for the design of educational and public health interventions tailored to students’ developmental characteristics.

## 5. Conclusions

This systematic review analyzed 29 studies examining the influence of PA performed before, during, and after the school day on positive psychology variables (well-being, resilience, subjective vitality, and mental toughness) in children and adolescents. Overall, the available literature suggests generally favorable relationships between school-related physical activity and these psychological outcomes, although the direction and strength of the findings varied across outcomes, activity characteristics, contexts, and study designs.

Interventions implemented during school hours, such as active lessons or active breaks, were found to consistently enhance emotional self-regulation, self-efficacy, and mood. Pre-school PA positively influenced emotional readiness and subjective vitality, whereas post-school PA, particularly within organized or sport-based contexts, was associated with higher resilience, self-esteem, and stress regulation. However, the magnitude and consistency of these benefits vary depending on factors such as the type of activity, the role assumed by the student, the social and educational environment, and the degree of autonomy and participation. These findings underscore the importance of designing comprehensive programs that incorporate appropriately dosed physical stimuli throughout the school day, tailored to the developmental characteristics of the student population. Nevertheless, these recommendations should be interpreted with caution because the evidence base includes heterogeneous study designs, intervention characteristics, outcome measures, and contextual conditions, which limit direct comparisons and the generalizability of the observed effects.

## Figures and Tables

**Table 1:** Database search strategy.

Database	Search Strategy	Limits	Filtered Elements
Scopus	(“physical active program” OR “physical activity academic lessons” OR “physically active classes” OR “physical education” OR “active break” OR “active lesson break” OR “active pauses” OR “recess” OR “recreation” OR “cooperative games” OR “active movement” OR “active displacement” OR “active pre-school programs” OR “extracurricular sport” OR “after school activities” OR “extracurricular activities”) AND (“wellbeing” OR “resilience” OR “subjective vitality” OR “mental toughness”) AND (“children” OR “child” OR “childhood” OR “students” OR “adolescent” OR “adolescents” OR “school” OR “secondary school”)	Articles published between 1 January 2014 and 31 May 2026 Age: 6–18 years Language: English and Spanish Journal article	38
PubMed			997
Web of Science			45
Eric			202

Note: This table shows the search strategy that has been used to find the articles.

**Table 2:** Checklist scores of included studies.

	Authors	A	B	C	D	E	F	Total Score	Checklist Score Range
1	Abbasi et al. [72]	2	2	2	2	2	0	10	9–12
2	Alshallal et al. [73]	2	2	2	2	1	2	11	9–12
3	Altunkurek & Bebis [74]	2	2	2	2	2	2	12	9–12
4	Bakir & Kangalgil [75]	2	2	2	2	2	0	10	9–12
5	Bell et al. [76]	2	2	2	2	2	2	12	9–12
6	Brand et al. [77]	2	2	2	2	2	2	12	9–12
7	Costigan et al. [78]	2	2	2	2	2	2	12	9–12
8	Griciūtė, [79]	2	2	2	2	2	0	10	9–12
9	Guillamón et al. [80]	2	2	2	2	2	2	12	9–12
10	Guo & liang [37]	2	2	2	2	2	0	10	9–12
11	Happ et al. [81]	2	2	2	1	2	0	9	9–12
12	Ho et al. [55]	2	2	2	2	2	2	12	9–12
13	Költő et al. [44]	2	2	2	2	2	2	12	9–12
14	Korcz et al. [82]	2	2	2	2	2	2	12	9–12
15	Li et al. [83]	2	2	2	1	2	2	11	9–12
16	Madsen et al. [84]	2	2	2	2	2	2	12	9–12
17	Mavilidi et al. [38]	2	2	2	2	2	2	12	9–12
18	Moljord et al. [85]	2	2	2	2	2	2	12	9–12
19	Moore at al. [50]	2	2	2	2	2	0	10	9–12
20	Ramírez-Granizo et al. [57]	2	2	2	2	2	0	10	9–12
21	Riches et al. [46]	2	2	1	1	2	0	8	5–8
22	Ruiz-Ariza et al. [86]	2	2	2	2	2	2	12	9–12
23	Ruiz-Ariza et al. [87]	2	2	1	2	2	2	11	9–12
24	Schmidt et al. [88]	2	2	2	2	2	2	12	9–12
25	Sheng et al. [89]	2	2	2	2	2	2	12	9–12
26	Silva et al. [90]	2	2	2	2	2	2	12	9–12
27	Smedegaard et al. [91]	2	2	2	2	2	2	12	9–12
28	Wade et al. [92]	2	2	2	2	2	0	10	9–12
29	Zou & Liu [93]	2	2	2	2	2	2	12	9–12

Note. Checklist score ranges were used as operational descriptive categories only: 9–12, 5–8, and 0–4. These categories do not represent validated levels of internal validity or risk of bias. A = The study was a full peer-reviewed article. B = Conducted before, during, or after school hours. C = Clear description of PA and positive psychology variables (well-being, resilience, subjective vitality, and mental toughness). D = Sample composed of youth aged 6–18 years. E = Cross-sectional or longitudinal design with control group or condition. F = Data adjusted for potential confounders.

**Table 3:** Main characteristics of analyzed studies.

		Authors/Year/Title	Study Design/ Intervention (Duration)/ Confounders	Sample/Age (Years)/ Country	Groups/Physical Activity Measures/Intensity	Psychology (Variables) Measures	Results
CROSS-SECTIONAL STUDIES	Physical activity before school hours	Költő et al. [44]/Transport to School and Mental Well-Being of Schoolchildren in Ireland	Cross-sectional design/Confounders: Gender, age, socioeconomic status and area of residence	9077 students/10–17 years/Ireland	Cross-sectional. IV: mode of transport to and from school. DV: Mental Well-being.	Mental Well-being: World Health Organization Five-item Well-being Index (WHO-5); Five-item Mental Health Inventory (MHI-5); The Cantril ladder	Those who reported using public transport reported poorer mental well-being than those using other means of transport, but adjusting for sociodemographic variables obscured these differences. The only exception was excellent health, where children who cycled outperformed the other three groups, even after adjustment for sociodemographic variables.
		Ruiz-Ariza et al. [86]/Influencia del desplazamiento activo sobre la felicidad, el bienestar, la angustia psicológica y la imagen corporal en adolescentes.	Cross-sectional design/Confounders: Age, BMI and gender	1012 students/14–15 years/Andalusia (Spain)	Cross-sectional. IV: Active travel. DV: Subjective Happiness, psychological well-being, psychological distress and body image.	Psychological Well-Being: General Well-Being Questionnaire	Adolescents who spent more minutes per day in active travel had higher levels of subjective happiness (β = 0.008, *p* = 0.020) and well-being (β = 0.010, *p* < 0.001), and lower psychological distress (β = −0.006, *p* = 0.026), independent of age, BMI and gender. No association or significant differences were found in any of the analyses with respect to body image (*p* > 0.05).
	Physical activity during school hours	Guillamón et al. [80]/Physical fitness and emotional well-being in school children aged 7 to 12 years	Cross-sectional design/confounders: Age	212 students/ 7–12 years/ Murcia (Spain)	Cross-sectional. IV: Physical fitness. DV: Emotional well-being.	Emotional well-being: Strengths and Difficulties Questionnaire (SDQ)	The results showed that those children with higher fitness (X ≥ P50) had more positive values in behavior problems (*p* = 0.002), hyperactivity (*p* < 0.001), problems with partners (*p* = 0.028), prosocial behavior (*p* < 0.001) and total score of difficulties (*p* = 0.029). Students with a lower level of total difficulties score (X < P40) had a better performance in 4 × 10 m (*p* = 0.010), manual dynamometry (*p* = 0.050), Course–Navette (*p* = 0.050) and general fitness (*p* = 0.040) with respect to those with medium and high levels. These results suggest a positive and bidirectional relationship between fitness and emotional well-being in primary school children.
		Li et al. [83]/How does physical activity improve adolescent resilience? Serial indirect effects via self-efficacy and basic psychological needs	Cross-sectional design/2 weeks/Confounders: Sex	1732 students/16–20 years/China	Cross-sectional. IV: PA. DV: Resilience.	Resilience: A modified Chinese version of the Connor-Davidson Resilience Scale	The results revealed that PA significantly and directly predicted resilience. Specifically, the positive relationship between PA and resilience was partially mediated by self-efficacy and basic psychological needs.
	After-school physical activity	Brand et al. [77]/During early to mid-adolescence, moderate to vigorous physical activity is associated with restoring sleep, psychological functioning, mental toughness and male gender	Cross-sectional design/Confounders: Gender	1361 students/11–16 years/Basel, Basel-Land, and Aargau (Switzerland)	Cross-sectional. IV: intensity of physical activity. DV: Subjective sleep/sleep disturbance, psychological functioning and mental toughness.	Subjective sleep: The Insomnia Severity Index (ISI) Psychological functioning: KID-SCREEN 52 Mental toughness: Mental Toughness Questionnaire (MTQ18)	The result showed that greater PA was related to improved psychological functioning, better subjective sleep, and increased mental toughness. Compared to male participants, females reported less PA, poorer subjective sleep, and had lower psychological functioning and mental toughness scores.
		Ramírez-Granizo et al. [57]/El rol de la resiliencia en la asociación entre la actividad física deportiva y aspectos académicos en escolares.	Cross-sectional design/No confounders	320 students/10–12 years/Spain	Cross-sectional. IV: PA, academic variable. DV: Resilience levels.	Resilience: Connor-Davidson Resilience Scale (CD-RISC)	The results showed that those who were more physically active had more resilient characteristics than those who were not physically active.
		Korcz et al. [82]/The role of school functioning, physical activity, BMI, sex and age in building resilience among Ukrainian refugee children in Poland	Cross-sectional design/Confounders: BMI, peer support and teacher support	248 students/10–15 years/Poland	Cross-sectional. IV: PA. DV: Resilience.	Resilience: Child and Youth Resilience Measure	The findings suggest that school environment, including enjoyment of school and strong support from teachers, plays a significant role in building resilience in children. PA enhanced the resilience of girls, whereas a higher BMI negatively impacted it.
		Griciūtė, A. [79]/Optimal level of participation in sport activities according to gender and age can be associated with higher resilience: Study of Lithuanian adolescents.	Cross-sectional design/No confounders	2899 students/10–18 years/Lithuanian	Cross-sectional. IV: Participation in sport activities (PSA). DV: Resilience.	Resilience: Resilience Scale for Adolescents (READ)	Significant differences were observed that indicated that adolescents could have higher resilience in comparison with sedentary peers if they participated in sport activities in line with the optimal level of participation in sport activities for resilience according to their gender and age (effect sizes were rather small; Bonferroni correction (*p* < 0.0125) was applied. According to the research data, there are possible specific mechanisms of interaction between resilience and participation in sport activities, and also, each age and gender group could involve a relevant different optimal level of participation in sport activities when resilience of adolescents is higher compared to sedentary peers.
		Sheng et al. [89]/Association between sports participation and resilience in school-attending students: a cross-sectional study	Cross-sectional design/Confounders: Gender, School grade, self-reported height and weight, sibling status, family structure, parental education level, ethnicity, family socioeconomic status, geographic district and school attended	67,281 students/10–17 years/Shenzhen (China)	Cross-sectional. IV: Sport Participation. DV: Resilience.	Resilience: The 10-item Connor-Davidson Resilience Scale (CD-RISC-10).	Approximately 47.1% of the entire sample reported no sports participation, and the average resilience score was 24.7. The regression model analysis revealed that, in the entire sample, increased in sports participation was linked to higher resilience scores (odds ratio [OR] for 1–3 times per month: 1.20, 95%CI: 1.16–1.24; OR for 1–2 times per week: 1.38, 95%CI: 1.33–1.43; OR for 3 times or more per week: 1.72, 95%CI: 1.65–1.79). Analyses stratified by gender and school grade indicated that sports participation was consistently associated with greater resilience.
		Happ et al. [81]/A salutogenic approach towards children’s overall physical activities, coping behavior and resilience: a mediation analysis	Cross-sectional design/No confounders	554 students/6–19 years/Tyrol (Austria)	Cross-sectional. IV: Overall PA (Non-sport PA and sporting PA). DV: Coping behavior and resilience.	Coping behavior: The kids coping scale. Resilience: Child and Youth Resilience Measure	General PA (sport and non-sport activity) is positively associated with coping behavior, which in turn mediates its impact on children’s resilience. Non-sport activities, such as active play and active mobility, contribute as much as sports to the development of coping skills and resilience.
		Moljord et al. [85]/Physical activity, resilience, and depressive symptoms in adolescence.	Cross-sectional design/one day (45 min)/Confounders: age	1229 students/13–18 years/Mid-Norway	Cross-sectional. IV: PA, resilience. DV: Depressive symptoms.	Resilience: Resilience Scale for Adolescents (READ)	PA was positively associated with resilience factors and negatively associated with depressive symptoms, this relationship being stronger in girls than in boys. In girls, PA directly decreased depressive symptoms, while in boys its effect depended on the structured style. Resilience factors such as personal competence and family cohesion were related to lower depressive symptoms in both genders.
		Ho et al. [55]/Physical activity improves mental health through resilience in Hong Kong Chinese adolescents	Cross-sectional design/confounders: Socioeconomic status and gender	775 students/12–14 years/Hong Kong	Cross-sectional. IV: PA level. DV: Mental Well-being, self-efficacy and resilience.	Mental well-being: Mental Component Summary score (MCS-12). Resilience: Chinese version of Connor-Davidson Resilience Scale (CD-RISC)	The results showed that the PA level was significantly correlated with the adolescent’s mental well-being (r = 0.66, *p* < 0.001), self-efficacy (r = 0.21, *p* < 0.001), and resilience (r = 0.25, *p* < 0.001), but not with school connectedness (r = 0.05, *p* = 0.15) or family connectedness (r = 0.06, *p* = 0.13). After adjusting for potential confounders in the path model, the PA level was significantly associated with mental well-being (β = 0.52, *p* < 0.001), and resilience was the only significant mediator (β = 0.31, *p* < 0.001), which contributed to 60% of this relationship
LONGITUDINAL STUDIES	Physical activity before school hours	Riches et al. [46]/Park and Stride for Health and Wellbeing: Evaluation of a wayfinding intervention to promote active travel to school in Oxfordshire, UK	Longitudinal design/1 month/No confounders	181 students/4–11 years/Oxfordshire (England)	2 groups: EG (n = 110): “Park and Stride” signage routes were implemented. CG (n = 70): did not receive the intervention and continued to go to school as usual.	Well-being: interview with parents and students Only interviews and surveys.	The results showed that the Park and Stride intervention showed a modest increase in active transportation, increasing students’ PA and well-being.
	Physical activity during school hours	Bell et al. [76]/The relationship between physical activity, mental wellbeing and symptoms of mental health disorder in adolescents: a cohort study	Longitudinal design (RCT)/7 days/Confounders: age, gender, ethnicity, socioeconomic status, study school, number of daylight minutes, baseline symptoms of mental health disorder, sleep, number of friends, belonging to teams and clubs, smoking and drinking alcohol	928 students/12–13 years/Southwest England	2 groups: EG (n = 335): Moderate to vigorous exercise. CG (n = 335): Usual practice without receiving the intervention.	Mental Well-being: Warwick Edinburgh Mental Wellbeing Scale (WEMWBS) Mental health disorder: Strengths and Difficulties Questionnaire (SDQ)	The results showed no evidence of an association between PA or intensity (moderate to vigorous physical activity [MVPA]) and mental well-being or global symptoms of mental health disorder.
		Abbasi et al. [72]/Comparing the Effects of Play Therapy and Selected Sports Exercises on Self-Confidence, Physical Self-Efficacy and Mental Toughness in Children	Longitudinal design (semiexperimental design)/8 sessions (45 min)/No confounders	60 students/9–11 years/Murcia (Spain)	3 groups: EG1 (n = 20): Participated in a play therapy program that included activities such as role-playing, mindfulness games, games for the expression of emotions, relaxation techniques and puppet play. EG2 (n = 20): Participated in a program of selected sports exercises, taken from the ‘Spark’ program. CG (n = 20): Did not receive any intervention.	Mental toughness: Sports Mental Toughness Questionnaire	It was found that there is a significant difference between the three groups in the average self-confidence, mental toughness, and physical self-efficacy. The influence of selected sports exercises on self-confidence, mental toughness and physical self-efficacy of children was revealed to be more than that of play therapy.
		Smedegaard et al. [91]/Improving the well-being of children and youths: a randomized multicomponent, school-based, physical activity intervention	Longitudinal design (RCT)/9 months/Confounders: gender, age and family social class	3124 students/10–13 years/Denmark	2 groups: EG (n = 1562): “Move for Well-being in Schools” program. CG (n = 1562): continued with usual school practice.	Well-Being: KIDSCREEN-27 Quality of Life Questionnaire for Children and Adolescents	The study showed that school-based PA interventions improved psychosocial well-being, as well as enjoyment of PA and self-esteem.
		Ruiz-Ariza et al. [87]/The effect of cooperative high-intensity interval training on creativity and emotional intelligence in secondary school: A randomized controlled trial	Longitudinal design (RCT)/2 days/week (20 min), 16 weeks/Confounders: Age and BMI	184 students/12–16 years/Andalusia (Spain)	2 groups: EG (n = 90): performed the Cooperative High-Intensity Interval Training (C-HIIT) program. CG (n = 94): performed static stretching during the intervention time.	Well-being: Trait and Emotional Intelligence Questionnaire Short Form (TEIQue-SF)	The EG increased well-being and sociability factors after the C-HIIT program (both *p* < 0.001). More specifically, inactive adolescents in the EG showed significant improvements in comparison to the CG in creativity, well-being, and sociability (*p* = 0.028, *p* < 0.001, and *p* < 0.003, respectively). However, we did not find changes among active adolescents.
		Moore et al. [50]/Developing Wellbeing Through a Randomised Controlled Trial of a Martial Arts Based Intervention: An Alternative to the Anti-Bullying Approach	Longitudinal design (RCT)/1 day/week (50 min), 10 weeks/No confounders	283 students/12–14 years/New South Wales (Australia)	2 groups: EG (n = 125): received a 10-week intervention program based on martial arts. CG (n = 158): did not receive the intervention program.	Resilience: Child and Youth Resilience Measure (CYRM-28)	The martial arts-based intervention significantly improved resilience and self-efficacy in adolescents, with greater increases in individual capabilities, caregiver relationships and contextual factors, as well as academic, social and emotional self-efficacy. No significant changes were observed in emotional or behavioral problems.
		Wade et al. [92]/The impact of exercise environments on adolescents’ cognitive and psychological outcomes: A randomised controlled trial	Longitudinal design (RCT)/No confounders	90 students/15–16 years/Newcastle (Australia)	4 groups: EG1 (n = 21): Aerobic activities and body weight resistance exercises indoor. EG2 (n = 24): Aerobic activities and body-weight resistance exercises in a park. EG3 (n = 25): Aerobic activities and bodyweight resistance exercises in a nature. CG (n = 20): This group stayed indoors and worked on a school project.	Vitality: Subjective Vitality Scale	Linear mixed models were used to examine changes within and between groups. The indoor group increased sustained attention accuracy compared to the park group. There were no between-group differences in working memory. The indoor and nature groups increased cognitive arousal compared to control. The Park group improved in state-level vitality compared to control.
		Silva et al. [90]/Effects of a multi-component program to promote physical activity on mental health indicators in adolescents: A pilot randomized controlled study	Longitudinal design (RCT)/(20 min/day, 2 days/week, 12 weeks)/Confounders: Gender, age, parents’ level of education, height and body mass	55 students/12–15 years/Jacarezinho and Santo Antonio da Platina (Paraná)	2 groups: EG (n = 31): Combination of muscle-strengthening and aerobic exercise. CG (n = 24): No Combination of muscle-strengthening and aerobic exercise; Physical Education classes as usual.	Psychological well-being: KIDSCREEN-27	The results showed that there was a significant reduction in sleep score and anxiety and depressive symptoms in the IG post 12 weeks of intervention (*p* < 0.05). However, no significant changes in psychological well-being were observed in either group (*p* > 0.05).
		Madsen et al. [84]/The “11 for Health in Denmark” intervention in 10- to 12-year-old Danish girls and boys and its effects on well-being—A large-scale cluster RCT	Longitudinal design (cluster randomized controlled trial)/2 days/week (45 min), 11 weeks/Confounder: Age, BMI and sex	3061 students/10–12 years/Denmark	2 groups: EG (n = 2533): “11 for Health in Denmark” program. CG (n = 528): continued with usual school practice.	Well-being: KIDSCREEN-27	Intervention program had a positive effect on physical well-being in girls (IG: 48.6 ± 8.5 to 50.2 ± 9.3), whereas the improvement was not significant in boys. The program also had a positive impact on well-being scores for peers and social support (IG: 50.2 ± 10.2 to 50.8 ± 10.1), though when analyzed separately in the subgroups of boys and girls the changes were not significant. No between-group differences were found for psychological well-being or school environment.
		Mavilidi et al. [38]/Effect of a Time-Efficient Physical Activity Intervention on Senior School Students’ On-Task Behaviour and Subjective Vitality: the ‘Burn 2 Learn’ Cluster Randomised Controlled Trial	Longitudinal design (RCT)/2 days/week, 1 year/Confounders: Socioeconomic status, geographic location, educational advantage of the student population and subject area	221 students/16–18 years/New South Wales (Australia)	2 groups: EG (n = 114): high-intensity PA (B2L). CG (n = 107): continued with usual school practice.	Subjective vitality: Subjective Vitality Scale	The results showed that moderate vigorous PA improves high school students’ on-task behavior and subjective vitality.
		Schmidt et al. [88]/Changes in physical activity, physical fitness and well-being following a school-based health promotion program in a Norwegian region with a poor public health profile: A non-randomized controlled study in early adolescents.	Longitudinal design/7 months/Confounders: Age, gender, parental education level and BMI	644 students/13–15 years/Norway	2 groups: EG (n = 197): “Active and Healthy Kids” program. CG (n = 447): continued with usual school practice.	General Well-being: KIDSCREEN-27 questionnaire Subjective vitality: The Subjective Vitality Scale by Ryan and Frederick	There was a group x time effect on school-based PA (*p* < 0.05), but not total PA, as well as on physical fitness (*p* < 0.05) and vitality (*p* < 0.01), that is to say, the multi-component school health promotion program led to positive changes in school PA levels, physical fitness, subjective vitality and health-related quality of life.
	After-school physical activity	Alshallal et al. [73]/Total and temporal patterning of physical activity in adolescents and associations with mental wellbeing.	Longitudinal design (randomized-controlled trial Get Others Active (GoActive))/4 months/Confounders: age, sex, ethnicity, sleep, body fat and socioeconomic status	1983 students/13–14 years/Essex and Cambridgeshire (East of England)	IV: PA (GoActive Program). DV: Mental Well-being.	Mental well-being: The Warwick Edinburgh Mental Wellbeing Scale.	This longitudinal analysis showed positive associations between PA and later mental well-being in both male and female adolescents across most time segments.
		Guo & Liang. [37]/Physical activity as a causal variable for adolescent resilience levels: A cross-lagged analysis.	Longitudinal design (cluster sampling method)/1 year/No Confounders	818 students/12–17 years/Wuhan	IV: PA DV: Resilience No groups longitudinal observational design.	Resilience: The Adolescent Resilience Rating Scale	The results indicated there was a significant positive correlation between PA and adolescent resilience; PA had a significant predictive effect on adolescent resilience.
		Costigan et al. [78]/Associations between physical activity intensity and well-being in adolescents.	Longitudinal design (cluster randomized controlled trial)/15 months/Confounders: Gender, body mass index (BMI), ethnicity	1223 students/13–15 years/Western Sydney	2 groups: EG (n = 611): received the workshop training. EC (n = 612): did not receive training.	Well-being: Positive and Negative Affect Scale (PANAS)	Light and moderate PA was not associated with well-being. Higher levels of vigorous PA were associated with more positive affect [β(SE) = 0.307 (0.06), *p* < 0.001], to an estimated vigorous PA turning point [Point(95%CI) = 36.48 min/day (31.39–41.59)]. Similarly, higher levels of vigorous PA were associated with less negative affect [β(SE) = −0.250 (0.06), *p* < 0.001] up to the estimated vigorous PA turning point [Point(95%CI) = 37.35 min/day (31.27–43.44)].
		Bakir & kangalgil. [75]/The Effect of Sport on the Level of Positivity and Well-Being in Adolescents Engaged in Sport Regularly.	Longitudinal design/2 days/week (1 h), 10 weeks/No confounders	60 students/15–17 years/Turkey	3 groups: EG1 (n = 20): regular and scheduled sporting activities, including training and competitions. EG2 (n = 20): regular social activities, such as cinema, theater, sightseeing, picnics, etc. CG (n = 20): continues to go about their daily lives without participating in scheduled sporting or social activities.	Psychological well-being: “Warwick-Edinburgh Mental Well-being Scale” (WEMWBS) scale	Based on the study results, significant differences between pre-test and posttest positivity were identified. There were significant differences in mental well-being between the sports activities group and the control group.
		Altunkurek & Bebis. [74]/The effects of wellness coaching on the wellness and health behaviors of early adolescents.	Longitudinal design (RCT)/1 day/week (90 min), 12 weeks/Confounders: Gender, BMI, family and friend relationship perception, participation in physical education, family type and education level	132 students/12–15 years/Turkey	3 groups: EG1 (n = 33): received a comprehensive wellness coaching program that included PA, individual interviews and group education. EG1 (n = 33): received group education but without the PA components and individual coaching. CG (n = 66): did not receive any intervention.	Well-being: The Five-Factor Wellness Scale—Adolescent Form (5F-Wellness-AF)	The data indicated a significant difference in the total 5F-Wellness-AF and ALPS scores after the program. No statistically significant difference was observed in the total scores of the two scales for the Health Education Group, while the Control Group’s 5F-Wellness-AF mean score declined after the intervention. At the beginning of the study, 15.2% of students in the wellness coaching program were overweight; this percentage decreased to 9.1% at the end of the study.
		Zou & Liu [93]/Promoting resilience in adolescents: the role of school physical activity	Longitudinal design (RCT)/3 day/week (60 min), 12 weeks/Confounders: Age and gender	200 students/12–17 years/Chengdu (China)	2 groups: EG (n = 100): conducted a PA program that included team games, individual exercises and group reflection. CG (n = 100): conducted a program of non-physical activities that included board games.	Resilience: Chinese version of the Connor-Davidson Resilience Scale (CD-RISC)	The physically active group experienced a significant increase in resilience, but the control group showed no significant change in resilience.

Abbreviation: PA = Physical Activity.

**Table 4:** Recommendations for physical activity before, during and after school time to produce short and long-term effects on well-being, resilience, subjective vitality and mental toughness in children or adolescents.

Time and Type of Physical Activity	Reference Program	Type/Duration/Intensity/Examples of Activities (Ordered from Least to Most Difficult)	Effects on Well-Being, Resilience, Subjective Vitality or Mental Toughness
Physical Activity Before School Hours			
Physical Activity Before School Hours/Aerobic and Strength-Based Physical Activity with a Playful–Cooperative Component	Build Our Kids Success (Boks) [129]	Running, obstacle courses, and weekly skill-based activities/60 min per day, 3 days per week/Moderate intensity Partner planks (students lie face down facing their partner, supporting themselves on their forearms and toes, keeping the body straight like a plank for 20 s without letting the abdomen drop). Obstacle relay race (students are divided into two groups and must run to a designated area while overcoming obstacles such as jumping over cones or weaving in a zigzag pattern. Upon returning, they tag the next teammate to start. The first team to complete the circuit wins). Group jumping jacks (students form a circle and perform jumping jacks together, jumping with legs apart and arms raised above the head, then jumping again to return to the initial position, repeating continuously).	These activities promote psychological well-being (e.g., the relay race) and strengthen resilience and mental toughness (e.g., partner planks) by requiring sustained effort, self-control, and teamwork.
Physical Activity Before School Hours/Playful, Cooperative, and Technical–Motor Aerobic Activity	TELUS Active in the AM [130]	Structured cooperative games and technical circuits/90 min per day, 2 days per week/Moderate to vigorous intensity Capture the flag (to practice dodging and running among moving obstacles. The goal is to capture the opposing team’s flag and bring it back to one’s own base to win the game). Zigzag dribble and basketball shooting (students form groups and complete a zigzag course quickly, followed by a basketball shot at the hoop). Mini-basketball match (teams play against each other to apply the previously learned skills).	Dynamic and cooperative activities such as games, circuits, and team sports enhance psychological well-being by generating enjoyment and positive emotions. They also increase subjective vitality by elevating energy and motivation.
Physical Activity During School Hours			
Physical Activity During School Hours/Physical Education Based on Functional Exercises	SELF-FIT intervention [131]	Fitness circuits with a playful component/20 min per day, 2 days per week/Moderate intensity Fitness dice (students roll a dice with different activities, jumping, burpees, push-ups, planks, sit-ups, jogging in place, skipping, and heel kicks and perform whichever activity appears). Plank with a clap (students hold a plank position facing a partner and clap hands while maintaining body alignment without dropping). Stretch and relax (students perform dynamic movements involving large muscle groups, such as arm swings, squats with overhead stretches, or lunges combined with trunk rotation).	These activities foster psychological well-being and subjective vitality by increasing energy, enjoyment, and engagement. They also strengthen resilience and mental toughness through shared effort, self-control, and the overcoming of physical challenges.
	Martial Arts–Based Intervention Program [132]	Martial and self-defense skills/50–60 min per day, 1 day per week/Moderate to vigorous intensity Karate circuit (students work in small teams, rotating through stations to practice karate skills—strikes, blocks, kicks, stances—offering feedback and encouragement to teammates, and setting small team challenges to enhance motivation and collaboration). Self-defense drills (students practice defensive techniques in pairs or small groups, alternating attacker and defender roles, coordinating in multi-attacker scenarios to maintain calmness and collective strategy). Meditation (deep-breathing exercises combined with gentle dynamic movements, such as raising arms while inhaling and lowering them while exhaling, to enhance concentration and activate the body). Inner Warrior Meditation (short sessions of controlled breathing with strong, focused movements, such as forward punches or strength poses in karate stance, to promote self-control and mental resilience under pressure). Sparring (students engage in a tai chi-inspired “sticky hands” exercise emphasizing fluidity, control, and mutual awareness).	These activities significantly enhance resilience by promoting cooperation, self-control, and positive coping. They also increase well-being and subjective vitality through mindful, dynamic movement, and develop mental toughness by confronting physical and emotional challenges in a safe, collaborative environment.
Physical Activity During School Hours/High-Intensity Active Breaks	Burn 2 Learn [38]	High-Intensity Interval Training (HIIT)/20 min per day, 2 days per week/Vigorous intensity Animal jump rope (students jump rope in groups while imitating animal sounds indicated by the teacher, synchronizing to maintain rhythm; leadership roles rotate to foster cooperation). Zigzag ball run (teams dribble a ball while running through a zigzag circuit; the team who completes the circuit first wins). Running in place (students jog in place according to the teacher’s intensity cues). Tabata routine (for example 20 s of intense work followed by 10 s of rest).	These HIIT-style activities promote improvements in psychological well-being and subjective vitality by boosting perceived energy and reducing distress. The physical effort, coordination, and teamwork required also strengthen mental toughness by helping students manage brief, high-intensity challenges.
Physical Activity During School Hours/Physically Active Learning and Integrated Active Breaks	“Active and Healthy Kids” intervention [88]	Cooperative sports and games/45 min per day, 3 days per week/Moderate to vigorous intensity Math stations (students run to different stations and solve math problems in teams). Who am I? (groups use body movement to represent concepts or historical figures for others to guess). Jump counting (students count aloud in a foreign language while jumping or walking around the classroom). Dance for all (students imitate the dance shown by the teacher, then take turns imitating one another’s moves).	These activities enhance emotional well-being, motivation, and subjective vitality. Moreover, their cooperative nature contributes to the development of resilience and mental toughness.
Physical Activity During School Hours/Structured Active Breaks and Recess Periods	Daily physical activity integrated into the school day [133]	Guided circuits and activities/20 min per day, 5 days per week/Moderate to vigorous intensity Active breaks: Spelling jacks (students work in small groups, coordinating to jump in rhythm while spelling a teacher-assigned word. They take turns calling out letters and encouraging peers to maintain synchronization, fostering mutual support and teamwork). Walking breaks (students walk together in groups, engaging in light conversation or brief interactive tasks, such as sharing anecdotes, quick questions, or “Who am I?” games). Synchronized jumping jacks (students work in pairs or small groups to perform jumping jacks in unison, maintaining pace and movement coordination while motivating each other to gradually increase speed). Active recess: Warm-up walk (students walk around the field in small groups, following teacher prompts, “arms forward,” “knees high,” etc., while keeping synchronized movement and supporting peers in maintaining posture and rhythm). Carrot harvest (students are divided into teams to “harvest” carrots (objects scattered across a delimited area) by dividing tasks such as running, jumping, or crawling to collect as many as possible within a set time). Yoga style (students form a circle and perform a dynamic yoga sequence with fluid transitions between poses (sun salutation, warrior I and II, planks with lifts, and balance poses) guided by the teacher at a continuous, moderate-to-vigorous pace to promote strength, flexibility, and cardiovascular endurance).	These structured breaks and active recess periods promote psychological well-being and subjective vitality by reducing stress, improving mood, and increasing energy levels. They also strengthen resilience by integrating movement, cooperative play, and emotional regulation within positive social contexts.
Physical Activity After School Hours			
Physical Activity After School Hours/Multicomponent Programs with Playful and Holistic Physical Development Orientation	Team and individual games [93]	Team games and individual endurance and strength exercises/60 min per day, 3 days per week/Moderate to vigorous intensity Team obstacle challenge (teams complete an obstacle course that requires coordination and teamwork to overcome). Core battle (teams of 3–4 maintain isometric strength positions (plank, wall sit, superman) while the opposing team tries to distract them with light challenges, without physical contact. The team that maintains balance the longest wins). Mini-soccer match (teams play short soccer matches to apply previously learned physical and cooperative skills).	These physical dynamics foster emotional and social well-being by integrating cooperation, shared effort, and enjoyment. They also develop resilience and mental toughness by exposing participants to physical and emotional self-regulation challenges in a team environment.
	Multilateral Educational Program [51]	Team-based games and activities, including cardiovascular endurance and flexibility exercises/90 min per day, 2 days per week/Moderate to vigorous intensity Yoga style (students perform a dynamic yoga sequence in a circle, including fluid transitions between poses such as sun salutation, warrior I and II, planks with lifts, and balance postures, guided by the teacher to maintain continuous movement). Bicytour (students complete a group cycling route, navigating collaboratively using a map with predefined instructions). The Chain (two students start by holding hands to form the initial chain and try to tag others, who then join the chain by holding hands. The game continues until all participants are linked).	These activities promote resilience by integrating collaborative physical challenges that strengthen personal, emotional, and social resources. They also enhance well-being and subjective vitality by fostering enjoyment, group connection, and a sense of achievement.

## Data Availability

No new data were created or analyzed in this study. Data sharing is not applicable to this article.

## Supplementary Materials

The following supporting information can be downloaded at: https://www.mdpi.com/article/10.3390/healthcare14162625/s1. Table S1. Main characteristics of analyzed studies.

## Abbreviations

The following abbreviations are used in this manuscript:

PA	Physical Activity
PE	Physical Education
